# Twist-driven separation of *p*-type and *n*-type dopants in single-crystalline nanowires

**DOI:** 10.1093/nsr/nwz014

**Published:** 2019-01-31

**Authors:** Dong-Bo Zhang, Xing-Ju Zhao, Gotthard Seifert, Kinfai Tse, Junyi Zhu

**Affiliations:** 1 College of Nuclear Science and Technology, Beijing Normal University, Beijing 100875, China; 2 Beijing Computational Science Research Center, Beijing 100193, China; 3 Theoretische Chemie, Technische Universität Dresden, Dresden D-01062, Germany; 4 Department of Physics, the Chinese University of Hong Kong, Hong Kong, China

**Keywords:** twist, codoping, nanowire, generalized Bloch theorem, bond orbital theory

## Abstract

The distribution of dopants significantly influences the properties of semiconductors, yet effective modulation and separation of *p*-type and *n*-type dopants in homogeneous materials remain challenging, especially for nanostructures. Employing a bond orbital model with supportive atomistic simulations, we show that axial twisting can substantially modulate the radial distribution of dopants in Si nanowires (NWs) such that dopants of smaller sizes than the host atom prefer atomic sites near the NW core, while dopants of larger sizes are prone to staying adjacent to the NW surface. We attribute such distinct behaviors to the twist-induced inhomogeneous shear strain in NW. With this, our investigation on codoping pairs further reveals that with proper choices of codoping pairs, e.g. B and Sb, *n*-type and *p*-type dopants can be well separated along the NW radial dimension. Our findings suggest that twisting may lead to realizations of *p*–*n* junction configuration and modulation doping in single-crystalline NWs.

## INTRODUCTION

Recent experiments have made unprecedented advances in the synthesis of doped Si nanowires (NWs) [1,2] and other semiconductor NWs [3–6], where *p*-type or *n*-type dopants can be readily incorporated during growth [2–4]. Doped NWs are important in many aspects, especially due to their appeal as building blocks for future nanotechnology [7,8]. In particular, codoping with separated *p*-type and *n*-type dopants provides ideal conditions for charge modulation, where ‘free’ electrons and holes are separated by the built-in electric field. This feature brings critical benefits such as substantial suppression of charge recombination and scattering with dopants, all crucial for NW-based *p*–*n* junctions [9–11]. Note that such a codoping configuration is different from the notion of codoping in the traditional sense, where *p*-type and *n*-type partially (or fully) compensated codoping is sometimes employed in TiO_2_ or GaN to achieve certain electronic properties or defect stability [12].

To achieve such a codoping configuration on the nanoscale, early attempts at doped NWs usually employ a core–shell structure made of different host materials, with the core material doped with one species and the shell material doped with another species [9–11,13–17]. Such heterogeneous structures usually require complicated controls during the crystal growth and may often lead to interfaces with a lot of detrimental defects and deep traps. Therefore, it is ideal to dope *p*-type and *n*-type dopants uniformly into a single NW and they may automatically separate and occupy different regions in the NW. However, the realization of codoping with well separated *p*-type and *n*-type dopants in single-crystalline NWs still remains elusive. Fundamentally, this is mainly due to the difficulty of separating *p*-type and *n*-type dopants in homogeneous materials, which demands an effective modulation of the dopants’ spatial distributions.

In bulk single crystal, a substitutional dopant is prone to occupying the atomic site inside the unit cell where the dopant matches best the electronic environment and atomic size of the host atom. As such, the doping formation energy is locally optimized [18]. However, due to the translational symmetry, a dopant does not intrinsically exhibit an occupation preference for a specific lattice site in a crystal. In other words, the distribution of dopants is essentially uniform throughout the crystal. Modulation with uniaxial, biaxial, or hydrostatic stress [19,20], widely employed to tune the incorporation of dopants in bulk, has little impact on this characteristic. As a consequence, for a codoped crystal, the *p*-type and *n*-type dopants prefer to form charge-neutral pairs due to the Coulomb attraction between them. On the other hand, in very thin NWs, dopants may adopt a non-uniform distribution over the NW radial dimension. For example, it has been proposed that dopants B and P prefer to stay at the NW core or surface due to the finite-size effect and surface effect [21–23]. However, such preference may not hold as the NW size increases [24]. Moreover, the stability of surface doping is also susceptible to the surface chemistry and reconstruction [25,26]. Controlling dopants with desired distributions, independent of size and surface effects, is an open problem of doping on the nanoscale.

In this work, we show that the radial distribution of dopants in single-crystalline SiNWs can be substantially modulated by twisting, a typical structural distortion widely explored in both 2D [27,28] and 1D [29–32]. Specifically, in a twisted SiNW, a dopant will prefer an atomic site around the NW core if its atomic size is smaller than that of the host atom; otherwise, it will stay adjacent to the NW surface. Such distinct behaviors of dopants are due to the twist-induced inhomogeneous shear strain as evidenced here in SiNWs by a bond orbital model that relies on *sp*^3^ hybrid atomic orbitals and the parameterizations of hoppings using Harrison’s rule [33]. Therefore, *n*-type and *p*-type dopants can be readily separated along the NW radial dimension if they have different atomic sizes. The resulting codoping configuration acts effectively as an alternative to the more complex coaxial NWs [9–11,13–17]. Further, the preferential occupation of dopants may also lead to a modulation doping mechanism [34,35]. These revelations are further supported by our quantum mechanical (QM) simulations of twisted SiNWs containing B, N, Sb, Ga, and As dopants, enabled by the generalized Bloch theorem [36,37] coupled with self-consistent charge density functional tight-binding (scc-DFTB) that offers a realistic QM description of interatomic interactions [38,39]. Here, it is worth highlighting that, to realize such a codoping configuration, SiNW is the material of choice because it can be doped as *n*-type and *p*-type easily and there are plentiful candidates in the choice of *p*-type and *n*-type dopants with suitable size differences.

## RESULTS

**Figure 1.**
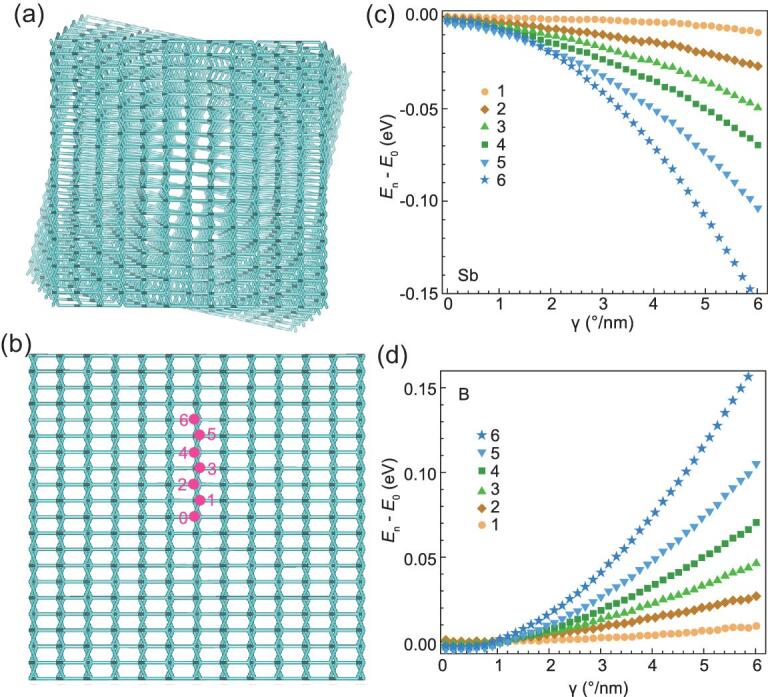
Axial view of the configuration of (a) twisted and (b) undistorted [113] SiNW with a diameter of ∼4 nm. Relative strain energy as a function of the twist rate γ of doped SiNW for (c) Sb and (d) B dopants at different atomic sites as indicated by filled circles in (b).

Mechanically, twisting induces an inhomogeneous shear deformation distributed along the NW radial dimension. To illustrate the geometric characteristic of a twisted NW under such a complex strain field, it is appropriate to use SiNWs as a representative system because Si is the most important semiconductor with a tetrahedral microstructure that is common in semiconductor materials; in addition, the stability and properties of doped SiNWs have been well investigated [1,2,25,26,40–42]. Figure 1a displays the twisted configuration of a [113] SiNW; the untwisted configuration is shown in Fig. 1b, which has a nearly square cross-section with a side length of ∼4 nm, a width where the size effect on the stability and mechanical property of SiNWs is negligible [43]. The four exposed surfaces— two {110} surfaces and two {111} surfaces—are all saturated with H atoms. This way, except atoms around the surfaces, an undistorted SiNW should maintain remarkably well the tetrahedral symmetry with vanishingly small deviations in bond length compared to bulk. Note that bulk Si has an equilibrium length of the σ bonds as *b*_0_ = 2.37 Å. For convenience, we adopt cylindrical coordinates to describe the geometry of the SiNW. In Fig. 1b, a series of atomic sites are highlighted. Consider an atom at site *n* (*n* = 1, …, 6) and initialize its coordinates as (*r*_0_, 0, 0) with $r_0=\sqrt{2/3}nb_0$; the coordinates of its nearest neighbors *i*, (*r*_*i*_, φ_*i*_, *d*_*i*_), located at the vertices of a tetrahedron, are analytically obtained; see Table S1 in the supplementary data.

**Figure 2.**
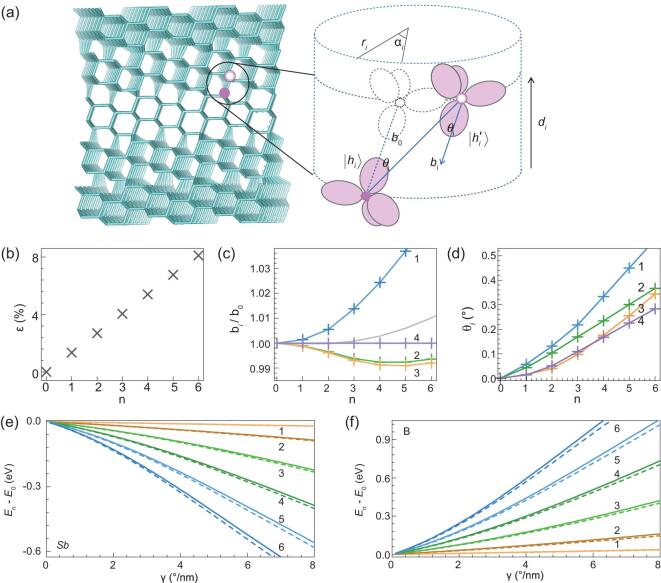
(a) Relative orientation of two *h*_*i*_ hybrids on a Si–Si bond in twisted SiNW. Before twisting, the *h*_*i*_ on the referred Si atom (solid circle) at (*r*_0_, 0, 0) and the hybrid $h_i^{\prime }$ on the neighbor *i* (open circle) at (*r*_*i*_, α_*i*_, *d*_*i*_) make a bond at a distance of *b*_0_. Under twisting, *h*_*i*_ and $h_i^{\prime }$ have a distance of *b*_*i*_ and adopt a misalignment angle θ_*i*_. (b) Shear strain ϵ of the twisted SiNW, (c) *b*_*i*_/*b*_0_, and (d) θ_*i*_ of the four nearest neighbors at twist rate γ = 4°/nm for different atomic sites, *n*, indicated in Fig. 1b. The thick gray curve in (c) is $\overline{b_i}/b_0$, an average of *b*_*i*_/*b*_0_ over different neighbors. Relative strain energy *E*_*n*_ − *E*_0_ of Sb-doped SiNW (e) and of B-doped SiNW (f) predicted by the bond orbital model for dopants at different atomic sites marked in Fig. 1b. Dashed curves denote the contribution from bond length variations.

In a twisted SiNW, for each atom of the structure, its four nearest neighbors no longer sit at the vertices of the ideal tetrahedron, involving tilting of the σ_*i*_ bond connecting this atom and its neighbor *i* (*i* = 1, 2, 3, 4) as well as variations in the length of the bond; see Fig. 2a. We indicate that such complex structural distortion can be properly delineated by mimicking the SiNW as a continuum object through a Cauchy–Born rule [44] using the small tetrahedron as a basic unit. Note that the degree of twist deformation of a NW can be characterized by a twist rate γ, defined as the twist angle per unit length along the NW axis. This way, for the atom at site *n*, the shear strain at twist rate γ is simply $\varepsilon =r_0\gamma =\sqrt{2/3}nb_0\gamma$, delineating the inhomogeneous distribution of the twist-induced shear strain in the NW radial direction, as shown in Fig. 2b. The corresponding variation of the σ_*i*_ bonds is evaluated by examining the distorted tetrahedron. The new bond length *b*_*i*_ and the tilting angle of the bond, θ_*i*_, are obtained to the second order of γ as

(1) \begin{eqnarray*} \left\{ \begin{array}{l} \displaystyle\left(\frac{b_i}{b_0}\right)^2\simeq 1+\frac{r_0r_i}{b_0^2}\left(2\tan {\phi _i}d_i\gamma +d_i^2\gamma ^2\right)\\ \\ \displaystyle\theta _i^2\simeq \left(\frac{4r_0^2r_i^2}{b_0^4}\tan ^2{\phi _i}+\frac{r_i^2}{b_0^2\cos ^2{\phi _i}}\right)d_i^2\gamma ^2.\end{array}\right. \end{eqnarray*}

As an example, Fig. 2 c and d shows the variation of *b*_*i*_ and θ_*i*_ with atomic sites *n* at twist rate γ = 4°/nm. Except for site *n* = 0, *b*_*i*_ is not common for different neighbors *i*: While *b*_*i*_ is elongated for neighbor *i* = 1, it is compressed for neighbors *i* = 2, 3. What is interesting is that $\overline{b_i}=1/4\sum _{i=1}^{4}b_i$, the average of *b*_*i*_ over the four nearest neighbors, is elongated; see Fig. 2c. This result hints that the average bond length of a NW increases with twisting.

Using these geometrical analyses as a basis, we are able to model the doping formation energy analytically by employing the bond orbital concept. We introduce the theory with the wavefunction partitioning in terms of orthogonal *sp*^3^ hybrids of tetrahedral Si, directed towards the nearest neighbors [33]:

(2) \begin{equation*} |h_i\rangle =\frac{1}{2}|s\rangle +\frac{\sqrt{3}}{2}|p\rangle ,i=1,2,3,4. \end{equation*}

The strong σ bonds are formed by the overlap of the *sp*^3^ hybrids on their nearest neighbors. Physically, such σ bonding nature is retained between the dopants and host Si atoms for dopants of a similar chemistry to Si. For dopant X, the bond length between X and Si, *b*_X_, may be different, as revealed by Table S2 in the supplementary data. In general, *b*_X_ > *b*_0_ if the atomic size of dopant X is greater than that of the Si atom; otherwise, *b*_X_ < *b*_0_.

Since the *sp*^3^ hybrids do not accommodate the twist distortion, the overlap between hybrids varies. The bond orbital theory offers an analytical approach to quantifying the variation of σ bond energy by adjusting the hopping integrals between hybrids *h*_*i*_. Combining the geometrical distortion, Eq. (1), we find the cohesive energy of a dopant X at site *n* as a sum of the bonding energy over nearest neighbors at a twist rate γ to the second order of *b*_0_/*b*_*i*_ and θ_*i*_ (see also the supplementary data):

(3) \begin{eqnarray*} E_\mathrm{X}(n)&=&\sum _i\langle h_i|H_n|h_{i}^{\prime }\rangle \nonumber \\ &\simeq&\sum _i \left( -\sqrt{(V-V^{\prime }\theta _i^2)^2\left(\frac{b_0}{b_i}\right)^4+V_p^2}\right.\nonumber\\ &&\left.+\,\frac{V^2\left(\frac{b_0}{b_i}\right)^4}{2\sqrt{V^2\left(\frac{b_0}{b_\mathrm{X}}\right)^4+V_p^2}}\right), \end{eqnarray*}

where $V=V_{ss\sigma }/2-\sqrt{3}V_{sp\sigma }-3V_{pp\sigma }/2=-8.79$ eV, and $V^{\prime }=-\sqrt{3}V_{sp\sigma }/2-3V_{pp\sigma }/2+3V_{pp\pi }/2=-7.51$ eV at the equilibrium bond length *b*_0_ according to Harrison’s universal scaling rule [33]. *V*_*p*_ is the polar energy between X and Si.

The relative strain energy of a twisted SiNW with dopant X at site *n* with respect to the case of X at site 0 is given as to the first order of *V*_*p*_/*V*:

(4) \begin{eqnarray*} E_{n}-E_0 &=&\left(E_\mathrm{X}(n)+E_\mathrm{Si}(0)\right)-(E_\mathrm{X}(0)\nonumber\\ &&+\,E_\mathrm{Si}(n)) \simeq \sum _i \left\lbrace \alpha _1\left[\left(\frac{b_0}{b_i}\right)^2-1\right]\right.\nonumber\\ &&\left.+\,\alpha _2\left[\left(\frac{b_0}{b_i}\right)^4-1\right]+\beta \theta _i^2 \right\rbrace ,\!\!\!\!\!\!\!\!\!\! \end{eqnarray*}

where $\alpha _1=V_p^2/2V$, $\alpha _2=1/2[(b_\mathrm{X}^2/b_0^2-1)V-(b_\mathrm{X}^6/b_0^6)(V_p^2/V)]$, and $\beta =V^{\prime }V_p^2/2V^2$. It is interesting to note that Eq. (4) is similar to the valence-force-field model [45,46], from which the physics behind the energetic responses of dopants to twisting can be revealed by exploring the behaviors of Eq. (4) for dopants with different atomic sizes. Considering X = Sb, B as examples [19], we have *V*_*p*_ = 0.65 eV for Sb–Si bonding, and *V*_*p*_ = 0.89 eV for B–Si bonding, and hence α_1_ = −0.03(−0.05) eV, α_2_ = 1.30( − 1.06) eV, and β = 0.03(0.04) eV for Sb (B) doping. With these parameters and Eq. (1) as input, the variation of *E*_*n*_ − *E*_0_ with γ reveals distinct energetic responses between Sb and B dopants. For Sb doping, *E*_*n*_ − *E*_0_ declines with γ, and is lower for atomic sites closer to the NW surface; see Fig. 2e. In contrast, for B doping, *E*_*n*_ − *E*_0_ increases with γ, and is higher for atomic sites closer to the NW surface; see Fig. 2f. These results reveal that dopants of greater atomic size, such as Sb, prefer sites near the surface while dopants of smaller atomic size, such as B, take sites adjacent to the core in a twisted SiNW.

More insight can be obtained by analyzing the contributions of different terms of Eq. (4). The dashed curves in Fig. 2e and f, representing the first two terms after the semi-equal of Eq. (4), reveal that the third term related to θ_*i*_ is negligible. The first term is also less important because α_1_ ≪ α_2_. Thus, Eq. (4) can be further simplified as

(5) \begin{eqnarray*} E_{n}-E_0&\simeq& \sum _i4\alpha _2\left(1-{b_i}/{b_0}\right)\nonumber\\ &=&16\alpha _2\left(1-\overline{b_i}/b_0\right). \end{eqnarray*}

Because the sign of α_2_ depends essentially on the atomic sizes of dopants, e.g. α_2_ > 0 for Sb and α_2_ < 0 for B, the distinct behaviors of *E*_*n*_ − *E*_0_ for dopants with different atomic sizes originate from the overall bond elongation in a twisted NW, i.e. $\overline{b_i}/b_0\ge 1$ at arbitrary γ and *n*, as shown in Fig. 2c. We note that such responses of dopants are unavailable for the case of hydrostatic, biaxial, or uniaxial stress.

In summary, the bond orbital theory provides an approximate estimation of the cohesive energy of covalent systems, from which we evaluate the strain energy of twisted NWs. As a demonstration of the validity of the bond orbital theory, we also carry out atomistic QM simulations of twisted NWs. Note that such microscopic simulations are practically forbidden because a twisted NW is no longer compatible with the prescribed translational periodic boundary conditions in standard QM approaches. Here, we instead employ a generalized Bloch theorem scheme developed with scc-DFTB. In this special scheme, the infinitely long twisted NW is described with a primitive motif that contains the same *N*_0_ atoms inside the translation unit cell from a repetition rule consisting of a screw symmetry,

(6) \begin{equation*} {\bf X}_{\lambda ,l}={\bf R}^{\lambda }(\Omega ){\bf X}_{0,l}+\lambda {\bf T}, \end{equation*}

where **X**_0, *l*_ represents atoms inside the repeating motif and **X**_λ, *l*_ represents those atoms inside the replica of the repeating motif indexed by λ. The index *l* runs over the *N*_0_ atoms inside the motif. The axial vector **T** and rotation **R** of angle Ω around the axis of the NW delineate the screw operator **S**. The twist rate is obtained as γ = Ω/|**T**|. The electronic states under such helical boundary conditions are described in terms of generalized Bloch functions [36,37], with which the optimal NW geometry and energy can be obtained through full structural relaxation at arbitrary **T** and Ω.

With the generalized Bloch theorem, we have carried out DFTB simulations of doped SiNW under the twisting shown in Fig. 1a with dopants at different atomic sites. We focus on two set of dopants: B, N of smaller atomic sizes, and Sb, Ga of larger atomic sizes, compared to Si. We note that the relative strain energy defined in the bond orbital analysis, Eq. (4), can be similarly obtained by identifying the difference between the formation energy of doped SiNW with a dopant at atomic site *n*, *E*_*n*_, and that of doped SiNW with a dopant at position 0, *E*_0_, i.e. *E*_*n*_ − *E*_0_. Surprisingly, the outcomes display the same trends as revealed by the bond orbital theory; see Fig. 1c and d for Sb and B doping, and Fig. S4a and b in the supplementary data for Ga and N doping. We have also considered the dopant As as being of nearly equal size to Si. As expected, *E*_*n*_ − *E*_0_ does exhibit a definitive trend with γ; see Fig. S4c in the supplementary data.

**Figure 3.**
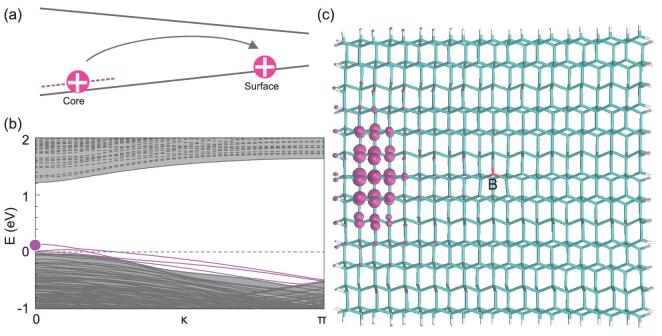
(a) Schematic band alignment of twisted SiNW. (b) Energy bands of γ = 4°/nm twisted SiNW with B doped at the NW core. (c) Distribution of the valence band maximum as indicated by the purple dot in (b).

**Figure 4.**
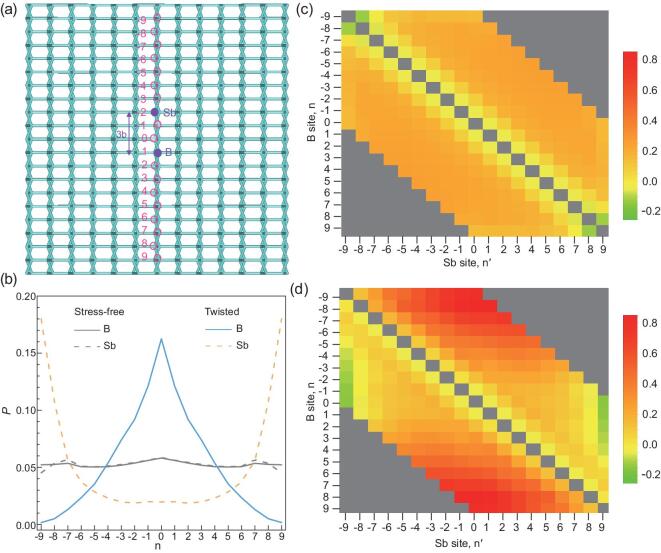
(a) Atomistic representation of a 3*b* Sb–B codoping configuration in SiNW. Integer numbers label the possible radial doping sites. (b) Occupation probabilities of B and Sb in codoped SiNW at γ = 7°/nm. The results for an undistorted SiNW are shown (gray) for comparison. Heat (*E*_*h*_ − *E*_1_) map of the relative formation energy of all possible codoping configurations calculated for (c) an undistorted and (d) a twisted (γ = 7°/nm) SiNW. Gray areas in the top-right and bottom-left corners indicate where data are not available. On the diagonal, having two dopants on the same site is physically forbidden.

The discovery of preferential occupations of dopants in twisted SiNWs leads to two important mechanisms. (i) Modulation doping. Due to the special deformation potential of Si [47], twisting a SiNW results in a reverse type I band alignment, as shown schematically in Fig. 3a. As such, a B dopant at the NW core will result in the formation of a hole state in the region near the NW surface. A supporting illustration of the charge distribution of the valence band maximum at κ = 0 shows separation between the charge and the dopant B of the twisted SiNW with B at the NW core; see Fig. 3b. (ii) Codoping. The distinct responses of dopants of different sizes to twisting hint at the possible separation of dopants. For codoped SiNW with Sb and B, we describe a codoping configuration with the separation between Sb and B in terms of the number of covalent bonds, *hb*. A 3*b* configuration is showcased in Fig. 4a. The stability of a codoping configuration *hb* is delineated by a relative formation energy *E*_*h*_ − *E*_1_, where *E*_1_ is the formation energy of a 1*b* Sb–B pair with B at site 0 and Sb at site 1. Each possible codoping configuration *hb* (*h* = 1, 2, …, 9) can be indexed with (*n*, *n*′), where *n* and *n*′ denote the atomic sites of B and Sb, respectively. To probe the impact of twisting on the codoping configurations, it is proper to employ some statistical approach. We have calculated *E*_*h*_ − *E*_1_ for all the possible configurations at γ = 0 and at γ = 7.0°/nm as summarized in Fig. 4c and d. Using these data as input, we calculate the occupation possibilities of B and Sb at different sites by assuming nearest-neighbor hopping with a transition probability of $\mathrm{min}\lbrace 1, \exp ^{-(E_h-E_1)/ k_\mathrm{B} T}\rbrace$; see the supplementary data for more details. Figure 4b shows that, in the undistorted SiNW, both B and Sb adopt a roughly uniform occupation distribution. As a result, there are no well defined codoping configurations with spatially separated B and Sb. In contrast, in the twisted SiNW, the occupation possibility of B reaches a maximum for sites near the NW core, while that of Sb has a maximum at sites approaching the NW surface. This observation indicates that the possibility of codoping configurations with large separations between B and Sb dopants are substantially enhanced by twisting.

## CONCLUSION

To conclude, our microscopic investigations reveal preferential occupation of dopants along the radial dimension in twisted SiNWs. A dopant of smaller atomic size than the host Si atom will prefer an atomic site near the NW core, while a dopant of larger atomic size than the Si atom will stay adjacent to the NW surface. Such distinct behaviors of dopants originate from the twist-induced radial inhomogeneous shear strain. The discussed twist distortion can be exerted on NWs by state-of-art experimental techniques. For example, utilizing the developed focused ion beam (FIB)-based irradiation [48–50], controllable twisting can be exerted on a SiNW; see the supplementary data for a possible experimental setup. We also note that similar twist deformation has been accomplished in more complex carbon nanotubes in a rotatable metal plate experimental setup [29,30]. The preferential site occupation of dopants hints at the possibility of single-crystalline SiNWs realizing modulation doping and codoping with separated *n*-type and *p*-type dopants, two novel mechanisms shown only in heterostructures before [9–11,34,35].

## METHODS

For the present study, microscopic simulations using standard methods are practically forbidden because a twisted NW is no longer compatible with the prescribed translational periodic boundary conditions via which standard electronic structure calculations are conducted. We thus employ a generalized Bloch theorem scheme instead. In this special scheme, to accommodate the helical boundary conditions of Eq. (6), the electronic subsystem is represented in terms of generalized Bloch functions,

(7) \begin{eqnarray*} \psi _{n,\alpha }(\kappa ,{\bf r})&=&\frac{1}{\sqrt{\zeta }}\sum _{\lambda =0}^{\zeta -1}\text{e}^{i\kappa \lambda }\sum _{\alpha {^{\prime }}}O_{\alpha \alpha {^{\prime }}}(\lambda )\varphi _{n,\alpha {^{\prime }}}\nonumber\\ &&({\bf r}-{\bf X}_{\lambda ,n}), \end{eqnarray*}

where $\varphi _{n,\alpha {^{\prime }}}({\bf r}-{\bf X}_{\lambda ,n})$ refers to the local atomic orbital α′ on atom *n* in the primitive cell rotated λ times. $\sum _{\alpha {^{\prime }}}O_{\alpha \alpha {^{\prime }}}(\lambda )\varphi _{n,\alpha {^{\prime }}}({\bf r}-{\bf X}_{\lambda ,n})$ is the symmetry-adapted orbital [36,37]. α runs over all the orbitals of a single atom. $O_{\alpha \alpha {^{\prime }}}$ are the matrix elements of **O**, rotating the local atomic orbitals by the azimuth angle of Ω. The phase factors and quantum number −π ≤ κ < π are obtained by imposing the screw boundary conditions. As the eigenfunction of the screw, ψ_*n*, α_(κ, **r**) forms a complete basis set involving only the *N* atoms in the primitive motif. The total energy is simply the sum of the eigenvalues of all the occupied electronic states. Usually, *N*_0_ is small, allowing comprehensive QM investigations, and the optimal NW geometry and energy are obtained through full structural relaxation at arbitrary **T** and Ω.

## Supplementary Material

nwz014_Supplemental_File
