# Supplementary material for: Twist-driven separation of p-type and n-type dopants in single-crystalline nanowires
Source: Natl Sci Rev. 2019 Jan 31;6(3):532–9. doi: 10.1093/nsr/nwz014 (PMC8291436; doi:10.1093/nsr/nwz014)
Supplement: nwz014_Supplemental_File [file nwz014_supplemental_file.pdf]

Twist-Driven Separation of p-type and n-type Dopants in Single Crystalline Nanowires

Dong-Bo Zhang[1,2,*], Xing-Ju Zhao[2], Gotthard Seifert[3], Kinfai Tse[4], and Junyi Zhu[4,*]
[1]College of Nuclear Science and Technology, Beijing Normal University, Beijing 100875, P.R. China
[2]Beijing Computational Science Research Center, Beijing 100193, P.R. China
[3]Physikalische Chemie, Technische Universitat Dresden, D-01062 Dresden, Germany
[4]Department of Physics, the Chinese University of Hong Kong, Hong Kong and
*Email: dbzhang@bnu.edu.cn; jyzhu@phy.cuhk.edu.hk

I. ATOMIC GEOMETRY OF SINW

Made from tetrahedral Si, the structure of the Si nanowire (SiNW) shown in Fig. 1(b) of the manuscript can be delineated by exploring the tetrahedron where one Si atom locates at the center with four nearest Si neighbors sit at the vertexes, Fig. S1. Let the Si atom sit at the tetrahedral center at $(r_0, 0, 0)$ in cylindrical coordinates, the coordinates of its four nearest neighbors can be analytically obtained, as summarized in Table S1. With these coordinates as a starting point, for a given Si atom at site n with $r_0 = \sqrt{2/3}nb_0$, the bond length variation and the tilting of bond angle of the strong σ_i bond connecting this atom and its neighbor i induced by twist can be derived. The results are summarized in Eq. (1) of the main text.

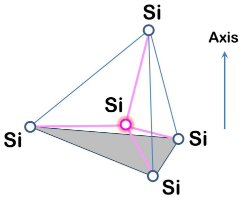

Figure S1. Schematic of the bonding network of tetrahedral Si with one Si atom at the tetrahedral center and four Si atoms at the vertexes following the crystallographic direction of the SiNW shown in Fig. 1(b) of the manuscript. The arrow shows the NW axial direction.

TABLE S1. Coordinates of the nearest neighbors of a referred Si atom at $(r_0, 0, 0)$ in the undistorted SiNW shown in Fig. 1(b) of the manuscript.

$i =$	1	2	3	4
r_i	r_0	$r_0 + \sqrt{\frac{2}{3}}b_0$	$r_0 - \sqrt{\frac{2}{3}}b_0$	r_0
ϕ_0	$b_0/3r_0$	$b_0/3(r_0 + \sqrt{\frac{2}{3}}b_0)$	$b_0/3(r_0 - \sqrt{\frac{2}{3}}b_0)$	$-b_0/r_0$
d_i	$2\sqrt{2}b_0/3$	$-\sqrt{2}b_0/3$	$-\sqrt{2}b_0/3$	0

II. CHOICE OF N-/P- DOPANT PAIRS

For codoping to be successful, defect solubilities of n- and p-type dopants are expected to be similar. This needs that the formation energies of both n- and p-type dopants are similar and small. We have calculated systematically the neutral defect formation energy for all group III and group V dopants following the methodology of [1] in a $3 \times 3 \times 3$ bulk Si supercell using density-functional theory as implemented in VASP [2] under the generalised gradient approximation to screen for candidate dopant pairs. The formation energy, calculated stress, and also the bond length between dopants and Si are summarized in Table S2. As expected, the doping induced stress increases down the group for both group III and V. Informally, this can be understood from the atomic sizes of dopants: larger the atomic size of a dopant, larger the stress of the doped bulk Si is. The formation energy does not show a simple trend as it depends also on the thermodynamic stability of secondary phases. In addition to the atomic size, the charge state also plays an important role to the formation energy. A p-type dopant tends to shrink the lattice while a n-type dopant tends to expand the lattice. Therefore, two pairs of $B + Sb$ and $In + N$ are most promising for codoping. In the present study, we consider B and Sb as dopants. With the charge state effect taken into account, B and Sb pair should be the most promising pair because B is a p-type and small-size element, while Sb is the n-type and large-size element.

In general, a dopant X makes bond with Si with a bond length, b_X, different from the Si-Si bond. In Table S2, we also list the bond length of b_X for different dopants.

III. BOND ORBITAL MODELING OF TETRAHEDRAL SI

We start with the common sp^3 hybrids directing toward the four nearest neighbors indexed with integer number $i = 1, 2, 3, 4$ [3],

$$|h_i\rangle = \frac{1}{2}|s\rangle + \frac{\sqrt{3}}{2}|p\rangle. \tag{S1}$$

The strong chemical bonds are formed by the overlap of the nearest neighbor sp^3 hybrids, $|h_i\rangle$, and the covalent

TABLE S2. Neutral defect formation energy (H_f), lattice stress (σ), and bond length between dopants and Si of group III and group V.

Group III			
	H_f (eV)	σ (kbar)	b_X ($\AA$)
B_{Si}	0.79	-3.87	2.09
Al_{Si}	0.92	1.02	2.43
Ga_{Si}	0.77	0.74	2.41
In_{Si}	1.68	2.55	2.56

Group V			
	H_f	σ	b_X
N_{Si}	1.47	-5.82	2.04
P_{Si}	0.18	-1.20	2.36
As_{Si}	0.41	-0.02	2.45
Sb_{Si}	0.98	1.88	2.58

energy per bond is evaluated as

$$\begin{aligned} E_{cov} &= 2\langle h_i|H|h_i^{'}\rangle \\ &= \frac{1}{2}V_{ss\sigma} - \sqrt{3}V_{sp\sigma} - \frac{3}{2}V_{pp\sigma}, \end{aligned} \tag{S2}$$

where, $V_{ss\sigma}$, $V_{sp\sigma}$ and $V_{pp\sigma}$ are hopping integrals between atomic orbitals on the nearest neighbors at the equilibrium bond length, b_0. For example,

$$V_{ss\sigma} = \langle s|H|s^{'}\rangle. \tag{S3}$$

IV. BOND ORBITAL MODELING OF TWISTED SINWS

Under twisting, the tetrahedral unit in a SiNW is distorted. Thus, for the σ_i bond connecting a Si atom at the tetrahedral center and its neighbor i, the bond length b_i varies and the bond adopts a tilting, θ_i, see Fig. 2(a). The variation of b_i and θ_i with twist rate γ is formulated in Eq. (1) of the main text. Neglecting the coupling between different hybrids on same atom, the binding energy per bond to the second order of θ_i,

$$\begin{aligned} E_{cov,i} = &\left(\frac{1}{2}V_{ss\sigma} - \sqrt{3}V_{sp\sigma}\cos\theta_i - \frac{3}{2}V_{pp\sigma}\cos^2\theta_i \right. \\ &\left. -\frac{3}{2}V_{pp\pi}\sin^2\theta_i\right)\frac{b_0^2}{b_i^2} \\ \simeq &(V - V'\theta_i^2)b_0^2/b_i^2. \end{aligned} \tag{S4}$$

where, $V = V_{ss\sigma}/2 - \sqrt{3}V_{sp\sigma} - 3V_{pp\sigma}/2$, and $V^{'} = -\sqrt{3}V_{sp\sigma}/2 - 3V_{pp\sigma}/2 + 3V_{pp\pi}/2$. The interatomic Hamiltonian matrix elements are derived from Harrison's universal scaling rule at the equilibrium bond length b_0 $V_{ss\sigma} = -1.80$ eV, $V_{sp\sigma} = 1.94$ eV, $V_{pp\sigma} = 3.03$ eV, and $V_{pp\pi} = -0.86$ eV [3]. Hence $V = -8.79$ eV and $V^{'} = -7.51$ eV. The factor of b_0^2/b_i^2 reflects the the variation of hopping elements following the Harrison's scaling law.

It is important to note that since E_{cov} obtained here is proportional to b_0^2/b_i^2. Thus, it has to be balanced by a repulsion if the tetrahedral Si structure is not to collapse. In general, this repulsive energy has a d_i^4 dependence. This can be understood by treating the coupling E_{cov} as arising from Coulombic potential energy, and the repulsive term as arising from kinetic energy. Then, at equilibrium, the potential energy will be twice of the kinetic energy according to the virial theorem [4]. With this argument, one can do a simple derivation and obtain the repulsive term. Thus, by properly setting the equilibrium bond length at b_0, the total binding energy per atom is updated as,

$$E = \sum_i (V - V'\theta_i^2)\frac{b_0^2}{b_i^2} + \frac{1}{2}V\frac{b_0^4}{b_i^4}. \tag{S5}$$

Indeed, it is straightforward to demonstrate that Eq. (S5) has a minima at $b_i = b_0$ and $\theta_i = 0$.

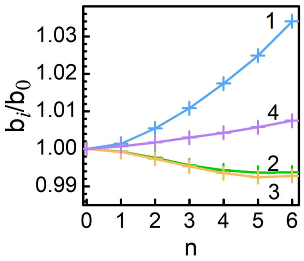

Figure S2. b_i/b_0 of the four nearest neighbors at twist rate $\gamma = 4°$/nm for different atomic sites, n, as shown in Fig. 1(b) of the manuscript.

V. BOND ORBITAL MODELING OF DOPED SINWS UNDER TWISTING

In a doped SiNW, to model the binding energy of the dopant atom which makes bonds with its four nearest Si neighbors, there are several aspects to be clarified. (i) In the stress-free SiNW, the equilibrium bond length between a dopant atom X and its Si neighbor, labeled as b_X, is generally different from the Si-Si bond length, b_0. Thus, those hopping elements obtained at bond length b_0 should be properly scaled to depict the binding energy of X-Si bonds. Hence, we updated $V \to Vb_0^2/b_X^2$, and $V' \to V'b_0^2/b_X^2$. (ii) For the polar X-Si bond, a polar energy, V_p, should be accounted for. This way, the binding energy per dopant atom in SiNW at twist rate γ is obtained as,

$$\begin{aligned} E_X(n) = &\sum_i \langle h_i|H_n|h_i^{'}\rangle \simeq \\ &\sum_i \left(-\sqrt{(V - V'\theta_i^2)^2\left(\frac{b_0}{b_i}\right)^4 + V_p^2} \right. \\ &\left. + \frac{V^2\left(\frac{b_0}{b_i}\right)^4}{2\sqrt{V^2\left(\frac{b_0}{b_X}\right)^4 + V_p^2}} \right). \end{aligned} \tag{S6}$$

This is Eq. (3) in the main text. To make the significance of Eq. (S6) more transparent, several aspects need to be clarified. (i) In general, $b_X \neq b_0$. The bonding energy of the σ bond between dopant X and Si is obtained following Harrison's scaling rule, as $V \to V(b_0/b_X)^2$ and $V^{'} \to V^{'}(b_0/b_X)^2$. (ii) The second term after the semi-equal sign reflects the repulsive interaction of electrons, which stabilizes the geometrical structure at the equilibrium bond length. Indeed, a simple derivation reveals that $E_X(n)$ has a minima at $b_i = b_X$ and $\theta_i = 0$.

(iii) The relaxation of the internal degree of freedom of the tetrahedron is not accounted for by the Cauchy-Born rule. However, an analysis of bond length variations in twisted SiNWs hints that this effect is not critical for the present problem. In the main text, for an atom at site n in the SiNW, the variations of the covalent σ_i bonds linking this atom and its four nearest neighbors are characterized by employing a Cauchy-Born rule [5] where the relaxation of the internal degrees of freedom of the tetrahedron is omitted. The outcome is summarized in Eq. (1) of the main text. To exemplify the relation revealed by Eq. (1),

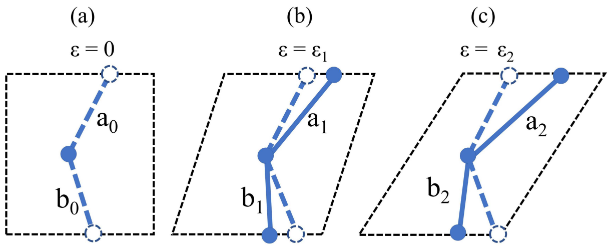

Figure S3. Schematic illustration of a three-atom molecule with two bonds made by nearest neighbors. (a) At equilibrium, one bond has a bond length a_0 and the other has a bond length b_0. Under shear deformation, at (b) $\varepsilon = \varepsilon_1$, bond lengths of these two bonds are a_1 and b_1, respectively, and at (c) $\varepsilon = \varepsilon_2$, bond lengths of these two bonds are a_2 and b_2, respectively. Note that $0 < \varepsilon_1 < \varepsilon_2$.

Fig. 2(c) of the manuscript plots out $b_i (i = 1, 2, 3, 4)$ at different atomic sites n.

As a comparison, Fig. S2 plots out $b_i (i = 1, 2, 3, 4)$ at different atomic sites n obtained with full scc-DFTB structural relaxation. Overall, one can see that the DFTB results compare well with the Cauchy-Born rule except some minors differences. This is supportive to the bond orbital modeling of the twisted SiNW.

VI. UNDERSTANDING OF THE BOND LENGTH VARIATIONS OF SINW WITH TWISTING

Fig. 2(c) of the manuscript plots out the bond lengths of those four bonds around the referred atom at different site n. One may wonder why some bonds are elongated while others are compressed and display nonmonotonic behaviors. Notice that the local shear strain in a twisted NW is proportional to n as shown in Fig. 2(b). Thus, to address this question, we need to understand the behaviors of the bond length variations under shear deformation. From this perspective, we indicate that the distinct behaviors of different bonds are due to their orientations.

As an illustration, we here use a planner three-atom molecule system as shown in Fig. S3. For the molecule shown in Fig. S3(a), two bonds are of equilibrium bond lengths a_0 and b_0, respectively. Under a shear strain $\varepsilon = \varepsilon_1$, due to the different orientations of these two bonds, it is easy to see that the a_0 bond is stretched to a_1 ($a_0 < a_1$) and the b_0 bond is compressed to b_1 ($b_0 > b_1$), Fig. S3(b). If we increase the shear strain to $\varepsilon = \varepsilon_2$, the a_0 bond is further stretched, and the b_0 bond is now also stretched from b_1 to b_2, Fig. S3(c). The behaviors of the a_0 bond and b_0 bond interpret the monotonic change of curve 1 and nonmonotonic changes of curves 2 and 3 in Fig. 2(c) of the manuscript.

VII. INTRODUCTION TO GENERALIZED BLOCH THEOREM

Quantum mechanical (QM) simulations are critical to deliver quantitative prediction of materials. However, simulations of twisted SiNWs pose a fundamental difficulty. This is because that twisting breaks the translational symmetry which the traditional atomistic approaches rely on. Here, we instead employ a generalized Bloch theorem scheme [6, 7] coupled with self-consistent charge density-functional tight binding (scc-DFTB) [8]. This special scheme adopts a screw symmetry to delineate the twist distortion. This way, the infinite long twisted NW can be fortunately described with a relatively small repeating motif which contains the same N atoms inside the translation unit cell,

$$\mathbf{X}_{\lambda,n} = \mathbf{R}^{\lambda}(\Omega)\mathbf{X}_{0,n} + \lambda\mathbf{T}, \tag{S7}$$

where, $\mathbf{X}_{0,n}$ represents atoms inside the repeating motif and $\mathbf{X}_{\lambda,n}$ represents those atoms inside the replica of the repeating motif indexed by λ. Index n runs over the N atoms inside the motif. Axial vector $\mathbf{T}$ and rotation $\mathbf{R}$ of angle Ω around the axis of NW defines the screw operator $\mathbf{S}$, and the twist rate is obtained as $\gamma = \Omega/|\mathbf{T}|$.

Special consideration is needed to accommodate the electronic subsystem to Eq. (S7). The one electronic states are represented in terms of generalized Bloch functions based on local basis,

$$\psi_{n,\alpha}(\kappa, \mathbf{r}) = \frac{1}{\sqrt{\zeta}} \sum_{\lambda=0}^{\zeta-1} \exp(i\kappa\lambda)\mathbf{S}^{\lambda}\varphi_{n,\alpha}(\mathbf{r})\chi(\sigma), \tag{S8}$$

where, $\varphi_{n,\alpha}(\mathbf{r})$ refers to the atomic orbital α on atom n and $\mathbf{S}^{\lambda}\varphi_{n,\alpha}(\mathbf{r})$ is the so-called symmetry-adapted orbital [9–11]. α runs over all the orbitals of single atom and n runs over all the N atoms inside the primitive motif. The phase factors and quantum number $-\pi \le \kappa < \pi$ are obtained by imposing the screw boundary conditions. χ represents the wavefunction for spin part with orientation σ in the collinear treatment.

More details are available in Ref. [6, 7], where we show the coupling of the generalized Bloch theorem coupled with self-consistent charge density functional tight-binding (scc-DFTB) [8, 12] that offers a realistic QM description of interatomic interactions. The generalized Bloch theorem provides a basis for the strain tunable property studies of quasi-one dimensional systems under inhomogeneous strain patterns. From the perspective of materials study, this theoretical framework is multipurpose. It is not only efficient for the calculation of the electronic property, but also useful to investigate structure, mechanics and other related properties. For example, with the generalized Bloch theorem calculation, we has revealed that in a zigzag graphene nanoribbons, an simple in-plane bending could induce spin-splitting and result in a half-metallic states [6].

VIII. RELATIVE FORMATION ENERGY OF C DOPED SINW UNDER TWISTING

In the main text, Fig. 1 shows the relative formation energy, $E_n - E_0$, of B and Sb doped SiNW under twisting, revealing distinct trends of the energetic stability of doped SiNW with respect to doping sites between B and Sb. We attribute such trends to a size effect of dopants: A dopant of smaller atomic size than the host Si atom (e.g., B)

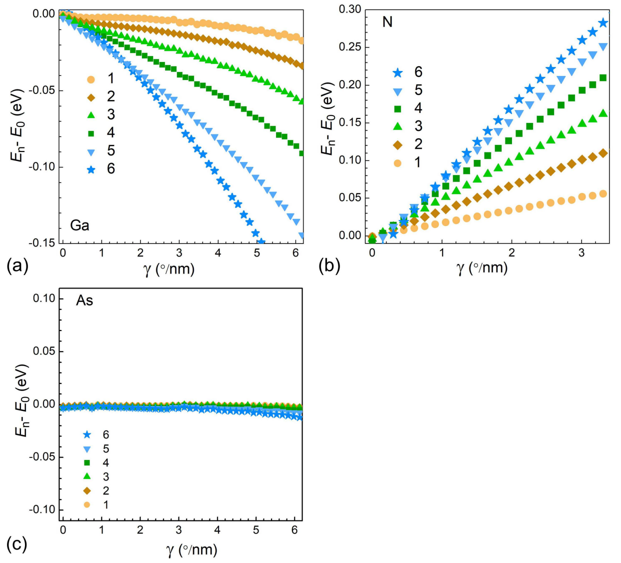

Figure S4. Relative strain energy as function of twist rate of doped SiNW for (a) Ga, (b) N, and (c) As dopants at different atomic sites as indicated by filled circles in Fig. 1(b) of the manuscript.

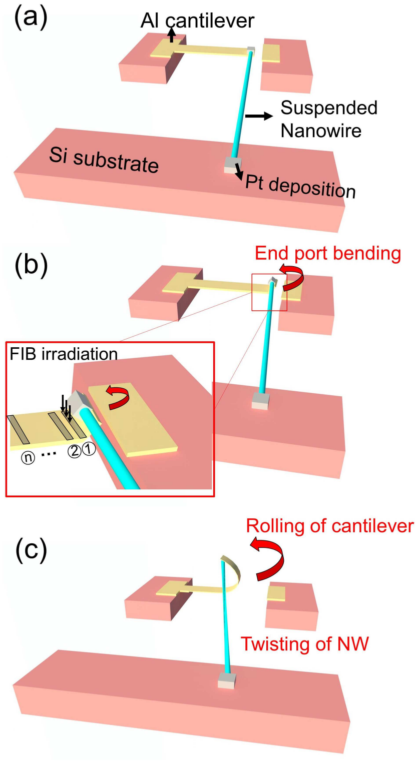

Figure S5. Schematics of the experimental setup using a focused ion beam (FIB) technique to twist a NW. (a) A suspended NW with one end welded to a terminal and the other end welded to a one-end fixed Al cantilever. (b) Ion radiation to the Al cantilever. A series of rectangular regions in grey are indexed with integer numbers (Insert). (c) Torsion of the NW induced by the curling of the Al cantilever.

prefers to stay near the NW core, while a dopant of larger atomic size (e.g., Sb) is prone to stay near the NW surface. Note that p-type dopant B is of smaller atomic size and n-type dopant Sb is of larger atomic size, compared to Si. Here, we consider p-type dopant Ga of larger atomic size and n-type dopant N of smaller atomic size than that of Si. $E_n - E_0$ shown Fig. S4(a) and (b) following the same trends. We also consider a n-type dopant As of a rough same atomic size with Si. As expected, $E_n - E_0$ shown Fig. S4(c) does not display a definitive trend.

IX. DOPANT PROFILE IN TWISTED VS. UNDISTORTED SI NANOWIRE

Here, we show the details of the calculation of the occupation probability of dopants in codoped SiNW. In Fig. 4(d) of the manuscript, we show a heat map of the relative strain energy of $E_h - E_1$ of different codoping configurations of the twisted SiNW. With this, we compute the occupation probability of dopant B and Sb at different site n of the considered SiNW. We adopt a nearest neighbour hoping approximation for both atoms, and the transition probability is evaluated via,

$$min(1, \exp^{-(E_h - E_1)/k_B T}), \tag{S9}$$

where, k_B is the Boltzmann constant, and temperature T is assumed to be 1100 K. Note that the separation of B and Sb dopants in SiNW can be obtained largely independent of temperature from $T = 300$ K up to 1500 K, covering typical temperatures of SiNW growth using various methods [13–16].

The probability is obtained from direct multiplication of the transition matrix raised to very large power with an even initial distribution in every configuration, the total probability of B and Sb at different sites is plotted in Fig. 4(b) of the manuscript. For the undistorted SiNW, both B and Sb have a roughly uniform occupation probability over all the atomic sites. This is because that although the formation energy has a significant increase (0.2-0.3 eV) with the distance between B and Sb, the nearest neighbour dopant B-Sb pair is slightly more favourable (-0.08 eV) near surface (see Fig. 4(c) of the manuscript), together with a slightly diminished transition probability near the surface due to geometry.

Under twisting with a twist rate of $7°/nm$, there is a destabilisation (stabilisation) of B (Sb) near surface and Sb (B) at the centre, with a maximum of 0.86 (-0.10) eV, Fig. 4(d) of the manuscript. This is reflected in the twisted dopant profile that B and Sb has a high probability of occupation at the centre and surface, respectively.

X. TWISTING A NANOWIRE: AN EXPERIMENTAL SETUP

Here we suggest a possible way to apply controllable twist distortion to a NW. The experimental setup is schematically shown in Fig. S5. In the experiment, a suspended nanowire (NW) with one end welded to a terminal and the other end welded to a one-end fixed Al cantilever is considered, Fig. S5(a) and the twist distortion to the NW is induced by the rolling of cantilever.

To obtain the cantilever, first of all, $\sim$ 100 nm thick Al

membrane was evaporated on Si substrate. Lithography and wet etching are then applied to define the pattern of cantilever. Next, isotropic dry etching was utilized to etch Si substrate to suspend Al thin film. Finally, the NW is transferred with one end welded to Si substrate and the other welded to the Al cantilever. Both ends of the NW are fixed by Pt deposition using focused ion beam-Chemical Vapor Deposition (FIB-CVD) and a FIB milling is used to cut one end of the cantilever.

The rolling process of the cantilever is based on the ion-radiation induced stress effect in thin films [17–19]. A rectangular region labeled with integer number, 1, [see grey areas in Fig. S5(b)] at the free end of the cantilever is first irradiated under ion beam irradiation, leading to the upward folding of the region 1 (the end portion) of the cantilever. The folding angle depends on the magnitude of the incident ion dose. Then, the second irradiation pattern is similarly set to the grey region 2. This leads a upward folding of grey region 2 and causes the region 1 to curl further. After a series of steps of irradiating ion to the cantilever from the free end to the fixed end, the Al cantilever is rolled up. Consequently, the NW is twisted, Fig. S5(c).

REFERENCES

1. Wei, S.-H. Overcoming the doping bottleneck in semiconductors. *Comput. Mater. Sci.* **2004**, *30*, 337–348.
2. Kresse, G.; Furthmüller, J. Efficient iterative schemes for ab initio total-energy calculations using a plane-wave basis set. *Phys. Rev. B* **1996**, *54*, 11169–11186.
3. Harrison, W. A. *Elementary Electronic Structure*; World Scientific, Singapore, 1999; pp 53–105.
4. Slater, J. C. The Virial and Molecular Structure. *The Journal of Chemical Physics* **1933**, *1*, 687–691.
5. Ericksen, J. L. *Phase Transformations and Material Instabilities in Solids*; Academic Press, New York, 1984; pp 61–77.
6. Zhang, D.-B.; Wei, S.-H. Inhomogeneous strain-induced half-metallicity in bent zigzag graphene nanoribbons. *Npj Comput. Mater.* **2017**, *3*, 32.
7. Yue, L.; Seifert, G.; Chang, K.; Zhang, D.-B. Effective Zeeman splitting in bent lateral heterojunctions of graphene and hexagonal boron nitride: A new mechanism towards half-metallicity. *Phys. Rev. B* **2017**, *96*, 201403.
8. Elstner, M.; Porezag, D.; Jungnickel, G.; Elsner, J.; Haugk, M.; Frauenheim, T.; Suhai, S.; Seifert, G. Self-consistent-charge density-functional tight-binding method for simulations of complex materials properties. *Phys. Rev. B* **1998**, *58*, 7260–7268.
9. White, C. T.; Robertson, D. H.; Mintmire, J. W. Helical and rotational symmetries of nanoscale graphitic tubules. *Phys. Rev. B* **1993**, *47*, 5485–5488.
10. Popov, V. N. Curvature effects on the structural, electronic and optical properties of isolated single-walled carbon nanotubes within a symmetry-adapted non-orthogonal tight-binding model. *New J. Phys.* **2004**, *6*, 17.
11. Allen, P. B. Nanocrystalline Nanowires: III. Electrons. *Nano Lett.* **2007**, *7*, 1220–1223.
12. Aradi, B.; Hourahine, B.; Frauenheim, T. DFTB+, a Sparse Matrix-Based Implementation of the DFTB Method. *J. Phys. Chem. A* **2007**, *111*, 5678–5684.
13. Schmidt, V.; Wittemann, J. V.; Senz, S.; Gosele, U. Silicon Nanowires: A Review on Aspects of their Growth and their Electrical Properties. *Adv. Mater. 21*, 2681–2702.
14. Das Kanungo, P.; Zakharov, N.; Bauer, J.; Breitenstein, O.; Werner, P.; Goesele, U. Controlled in situ boron doping of short silicon nanowires grown by molecular beam epitaxy. *Appl. Phys. Lett.* **2008**, *92*, 263107.
15. Tang, Y. H.; Sham, T. K.; Jurgensen, A.; Hu, Y. F.; Lee, C. S.; Lee, S. T. Phosphorus-doped silicon nanowires studied by near edge x-ray absorption fine structure spectroscopy. *Appl. Phys. Lett.* **2002**, *80*, 3709–3711.
16. Fukata, N.; Chen, J.; Sekiguchi, T.; Matsushita, S.; Oshima, T.; Uchida, N.; Murakami, K.; Tsurui, T.; Ito, S. Phosphorus doping and hydrogen passivation of donors and defects in silicon nanowires synthesized by laser ablation. *Appl. Phys. Lett.* **2007**, *90*, 153117.
17. Mao, Y.; Zheng, Y.; Li, C.; Guo, L.; Pan, Y.; Zhu, R.; Xu, J.; Zhang, W.; Wu, W. Programmable Bidirectional Folding of Metallic Thin Films for 3D Chiral Optical Antennas. *Adv. Mater.* **2017**, *29*, 1606482.
18. Chalapat, K.; Chekurov, N.; Jiang, H.; Li, J.; Parviz, B.; Paraoanu, G. S. Self-Organized Origami Structures via Ion-Induced Plastic Strain. *Adv. Mater.* **2013**, *25*, 91–95.
19. Park, B. C.; Jung, K. Y.; Song, W. Y.; O, B.-h.; Ahn, S. J. Bending of a Carbon Nanotube in Vacuum Using a Focused Ion Beam. *Adv. Mater.* **2006**, *18*, 95–98.
